# Immune-Specific Expression and Estrogenic Regulation of the Four Estrogen Receptor Isoforms in Female Rainbow Trout (*Oncorhynchus mykiss*)

**DOI:** 10.3390/ijms19040932

**Published:** 2018-03-21

**Authors:** Ayako Casanova-Nakayama, Elena Wernicke von Siebenthal, Christian Kropf, Elisabeth Oldenberg, Helmut Segner

**Affiliations:** 1 Centre for Fish and Wildlife Health, 3012 Bern, Switzerland; ayako0109@yahoo.com (A.C.-N); elena.wernicke@vetsuisse.unibe.ch (E.W.v.S.); christian.kropf@vetsuisse.unibe.ch (C.K.); Elisabeth.oldenberg@sunrise.ch (E.O.); 2 Department of Infectious Diseases and Pathobiology, Vetsuisse Faculty, University of Bern, Länggassstrasse 122, 3012 Bern, Switzerland

**Keywords:** estrogen receptor, isoforms, rainbow trout, immune system, reproductive cycle

## Abstract

Genomic actions of estrogens in vertebrates are exerted via two intracellular estrogen receptor (ER) subtypes, ERα and ERβ, which show cell- and tissue-specific expression profiles. Mammalian immune cells express ERs and are responsive to estrogens. More recently, evidence became available that ERs are also present in the immune organs and cells of teleost fish, suggesting that the immunomodulatory function of estrogens has been conserved throughout vertebrate evolution. For a better understanding of the sensitivity and the responsiveness of the fish immune system to estrogens, more insight is needed on the abundance of ERs in the fish immune system, the cellular ratios of the ER subtypes, and their autoregulation by estrogens. Consequently, the aims of the present study were (i) to determine the absolute mRNA copy numbers of the four *ER* isoforms in the immune organs and cells of rainbow trout, *Oncorhynchus mykiss*, and to compare them to the hepatic *ER* numbers; (ii) to analyse the *ER* mRNA isoform ratios in the immune system; and, (iii) finally, to examine the alterations of immune *ER* mRNA expression levels in sexually immature trout exposed to 17β-estradiol (E2), as well as the alterations of immune *ER* mRNA expression levels in sexually mature trout during the reproductive cycle. All four ER isoforms were present in immune organs—head kidney, spleen-and immune cells from head kidney and blood of rainbow trout, but their mRNA levels were substantially lower than in the liver. The ER isoform ratios were tissue- and cell-specific, both within the immune system, but also between the immune system and the liver. Short-term administration of E2 to juvenile female trout altered the *ER* mRNA levels in the liver, but the ERs of the immune organs and cells were not responsive. Changes of *ER* gene transcript numbers in immune organs and cells occurred during the reproductive cycle of mature female trout, but the changes in the immune ER profiles differed from those in the liver and gonads. The correlation between *ER* gene transcript numbers and serum E2 concentrations was only moderate to low. In conclusion, the low mRNA numbers of nuclear ER in the trout immune system, together with their limited estrogen-responsiveness, suggest that the known estrogen actions on trout immunity may be not primarily mediated through genomic actions, but may involve other mechanisms, such as non-genomic pathways or indirect effects.

## 1. Introduction

The main physiological function of estrogens in vertebrates is to regulate sexual development and reproduction. However, estrogens have pleiotropic functions and beyond the “classical” function in the reproductive axis, estrogens target a number of other physiological systems including the immune system [1]. In fact, for mammals it is well documented that estrogens like 17β-estradiol (E2) modulate the development, differentiation, life span, activation, and functioning of immune cells, and can have both immunostimulating and immunosuppressive actions [2,3,4,5]. The immunomodulatory activity of estrogens is a key proximate mechanism contributing to the known sexual dimorphism of mammalian immunity [6,7]. The primary effects of estrogens on the immune cells are mediated via rapid non-genomic signaling pathways as well as via the two nuclear estrogen receptor (ER) subtypes of mammals, ERα and ERβ [4]. Nuclear ER can either directly bind to estrogen response elements in gene promoters or serve as cofactors with other transcription factors such as nuclear factor-kappa beta (NFκB) [8]. ERα and ERβ are expressed in most cells of the myeloid and lymphoid cell lineages and in many hematopoietic progenitor cells [4,9,10,11]. The ratios of the two ER subtypes differ between immune tissues and cells, what has relevance for the diverse immunological effects of estrogens [12,13,14]. 

The immunomodulatory actions of estrogens in mammals vary with respect to target cell type, physiological condition of the organism or estrogen concentrations [2,3,15,16,17]. In particular, the female reproductive status and the associated changes of estrogen and ER levels have a major influence on the immune system response to estrogens [2]. With the evolution of internal fertilization and viviparity, mammals had to master a delicate balance between immunological protection of the mother against pathogens that are transmitted with fertilization, the prevention of immune responses against the spermatozoa, and immunological tolerance against the implantation of the semi-allogeneic embryos and the developing foetus [18,19,20]. In contrast to mammals, the reproductive strategy of lower vertebrates, such as teleost fish, relies on external fertilization and ovipary. Despite this difference, estrogens appear to have immunomodulatory actions in teleosts as well. A number of studies could show that immune parameters and immunocompetence of fish are influenced by estrogens, both by endogenous estrogens and by environmental (xeno) estrogens [21,22,23]. Moreover, recent research provided evidence that both membrane and nuclear ERs are expressed in immune organs and cells of teleosts [24,25,26,27,28,29,30,31]. In fact, the available evidence suggest that the immunomodulatory function of estrogens has been conserved throughout vertebrate evolution, despite the differences of reproductive strategies between oviparous and viviparous vertebrates [23].

The responsiveness of target cells to estrogens depends in large part on the cellular ratios of the various ER isoforms, their numbers and stability, and the regulation of ER activity and stability by the hormone signal, as well as by co-regulators and cross-talk with other signaling pathways [32,33,34,35]. While research during recent years has greatly advanced our understanding of the regulation of ER activity and turnover in mammalian cells [36,37,38] and how this drives the responsiveness of distinct cell types to estrogens, the current knowledge for teleost fish of the factors regulating ER activity and cell type-specific estrogen responsiveness is rather limited. With respect to the immune system of fish, information on absolute gene copy numbers of the ER in the immune organs and cells is lacking. Also, it is not clear yet whether piscine immune cells express all nuclear ER isoforms. Particularly for ERβ isoforms, there have been reports that they are not ubiquitously expressed in immune cells and organs [25,27,31,39]. Finally, while we have a reasonably good understanding of the autoregulation of the hepatic ERs in fish [35], no such database exists with respect to the estrogenic regulation of the ERs in the immune system. Given these knowledge gaps, the aims of the present study were to determine the absolute numbers of ER in immune organs and cells, and to compare them to the hepatic ER numbers, to analyse ER subtype ratios in the immune organs and cells, and to examine the alteration of immune ER expression levels in response to exogenous E2 and in association with the reproductive cycle. As experimental species, the rainbow trout, *Oncorhynchus mykiss,* was used. This species possesses four nuclear ER isoforms *ERα1*, *ERα2*, *ERβ1*, and *ERβ2*, which share a high degree of similarity of their amino acid sequences, particularly in the C-domain/zinc finger motif, in the activation function 1 (AF1) and AF2 domains [40]. In a first step, absolute gene copy numbers of the four *ER* were determined in the head kidney, the spleen, as well as in leukocytes that were isolated from the head kidney and from the blood of juvenile trout. In a next step, we aimed to gain insight into the regulation of the four ER subtypes in the immune system and examined the influence of exogenous E2 exposure on immune-specific ER profiles of juvenile rainbow trout, and we evaluated the immune *ER* mRNA profiles variation during the reproductive cycle and the associated fluctuations of endogenous levels of circulating E2 in mature female trout. 

## 2. Results

### 2.1. Absolute Gene Transcript Levels of Erα1, α2, β1, and β2 in Immune Organs and Cells of Juvenile Rainbow Trout in Comparison to Liver ER Gene Transcript Levels

**Figure 1:**
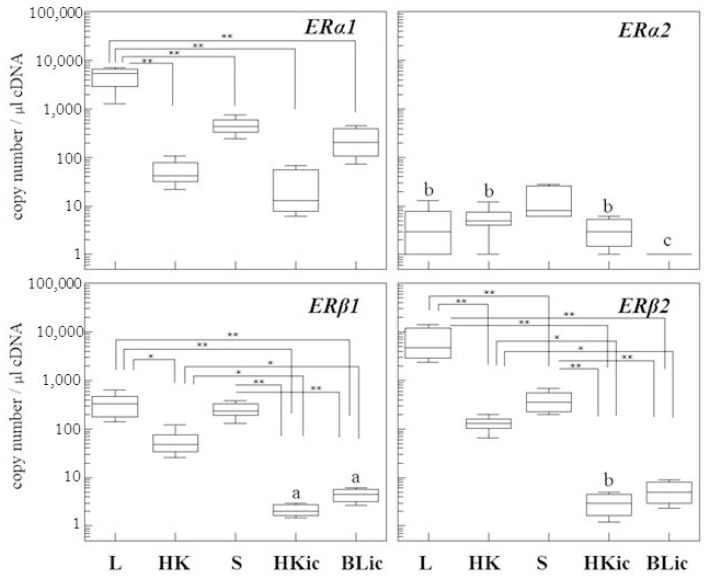
Absolute mRNA quantification of the four estrogen receptor (ER) isoforms in liver (L), head kidney (HK), spleen (S) and immune cells isolated from either head kidney (HKic) or blood (BLic) of 6-month-old female rainbow trout. The gene copy number of each isoform per 1 µL cDNA is presented by Box-Whisker plots (*n* = 5 individuals). Note logarithmic scale of y-axis. * *p* < 0.05, ** *p* < 0.01. a: under detection limit. b: Part of the sample was not detectable or under detection limit. c: not detected.

In juvenile rainbow trout, there exist distinct differences of the ER subtype ratios and profiles between the various organs and cells (Figure 1). Generally, the liver has significantly higher *ER* gene transcript levels than the immune tissues (except for *ERα2*). This applies particularly for *ERβ2*, where the liver gene transcripts are about 18 times higher than in the spleen, 55 times higher than in the head kidney and more than 1000 times higher than in the isolated leukocytes. Similar differences are observed for *ERα1*, with hepatic gene transcript levels being 10 times higher than in spleen and blood leukocytes, 160 times higher than in the head kidney, and 90 times higher than in the head kidney leukocytes. For *ERβ1* mRNA, expression levels in the liver are about 1.5 times higher than in spleen, six times higher than in head kidney, and about 80 times higher than in the isolated leukocytes, regardless whether they originate from the head kidney or the blood. In general, the mRNA lowest levels were found in the isolated immune cells (with the exception of *ERβ2*). 

When considering the mRNA profiles of ER isoforms for the various tissues and cells using *ERα1* mRNA as a reference point (Table 1), the *ERα1* isoform has slightly lower expression levels than *ERβ2* in liver and head kidney, equal levels in the spleen, and 10 to 110 times higher levels in the leukocytes. *ERβ1* mRNA levels have the greatest difference to *ERα1* mRNA in the blood leukocytes and the smallest in head kidney and spleen. *ERα2* is the isoform with the lowest mRNA expression levels, relative to *ERα1* mRNA, in all of the organs and cells of control animals. Thus, each organ and cell has a specific profile of the ER isoforms.

### 2.2. Changes of ER Gene Transcript Levels in Sexually Immature Juvenile Rainbow Trout Exposed to Exogenous E2

**Figure 2:**
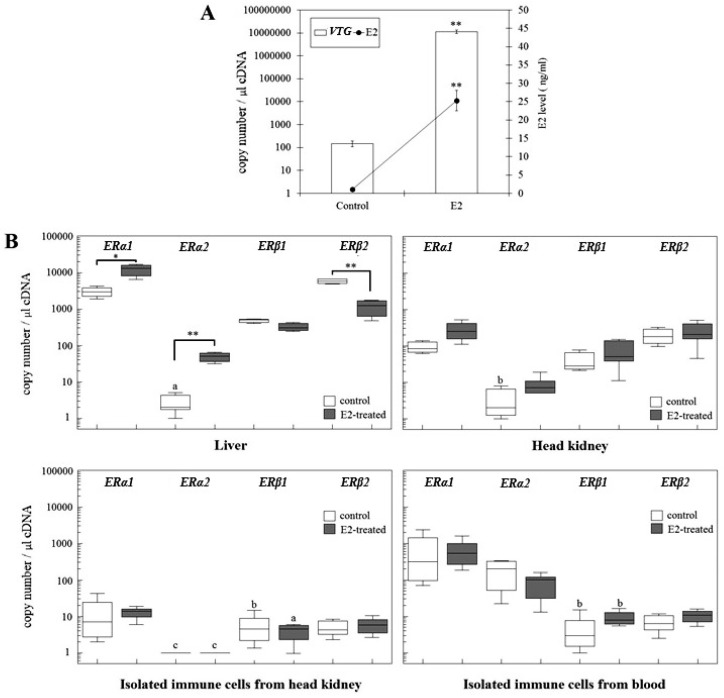
Response to exogenous 17β-estradiol treatment in 6-month-old juvenile female trout. Fish were fed with E2 containing pellets for five days and pellets prepared with only vehicle (ethanol) were used as control diets. (**A**). Absolute quantification of vitellogenin (VTG) mRNA in the liver and E2 levels in serum of the control (**C**) and E2-treatment (E2) groups. The absolute *VTG* gene copy number per 1 µL cDNA in the liver is shown as mean ± SE (*n* = 5 individuals). (**B**). Absolute mRNA quantification of the four ER isoforms in liver, head kidney, immune cells isolated from head kidney and blood of 6-month-old rainbow trout treated with E2. The gene copy number of each isoform per 1 µL cDNA is presented by Box-Whisker plots (*n* = 5 individuals). Control and E2-treated group were compared for statistical analysis. The asterisks denote statistically significant differences between control and E2-treated groups. * *p* < 0.05, ** *p* < 0.01. a: under detection limit. b: Part of the sample was not detectable or under detection limit. c: not detected.

Short-term (five days) exposure of sexually immature rainbow trout to E2 (via the diet) resulted in a significant elevation of plasma E2 concentrations and hepatic *VTG* gene transcript levels (Figure 2A), indicating that the treatment indeed induced an “estrogenic condition” in the animals.

The E2 treatment also affected the hepatic gene transcript levels of the two *ERα* isoforms: *ERα1* mRNA levels were significantly upregulated (4-fold) and those of *ERα2* mRNA even 17-fold (Figure 2B). In contrast, *ERβ2* was significantly downregulated, while *ERβ1* gene copy numbers showed no significant change. Interestingly, it was the *ERα2* isoform that showed the strongest E2 response among the hepatic ERs isoforms. In head kidney the E2 treatment remained without significant effects on the *ER* gene transcript levels, although there was a trend for elevated values, particularly for *ERα1*. Also, in the isolated immune cells, the estrogenic condition showed no significant effect on the ER expression levels, regardless whether the cells originated from the head kidney or the blood. Thus, the estrogenic condition had a prominent effect on the ER expression levels in the liver but did not clearly modulate ER expression in the immune system. 

**Figure 3:**
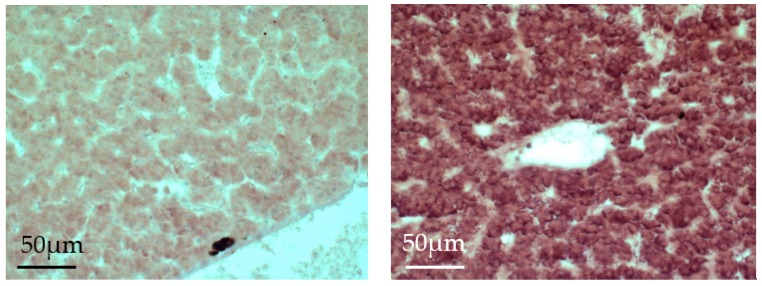
In situ hybridization of the *ERα1* mRNA in the liver of juvenile rainbow trout. Detection of the hybridization product was done using *ERα1* probes on liver sections of control (**left**) and E2-exposed (**right**) juvenile rainbow trout and detected with NBT-BCIP (dark-purple).

By means of *in situ* hybridization (ISH), we tried to visualize the cellular localization of the *ER*s mRNA in the immune organs of control and E2-treated fish. Liver tissue was used as control. We obtained a weak positive staining in the liver of control rainbow trout, and a very strong staining in the liver of E2-exposed trout (Figure 3). This finding is well in agreement with the RT-PCR results. In the immune organs, head kidney, and spleen, we did not obtain a positive staining result. Apparently, the sensitivity of the ISH was not sufficient to stain the low mRNA numbers of ERs in the immune organs.

### 2.3. Changes of ER Gene Transcript Levels in Sexually Mature Adult Rainbow Trout Females during the Reproductive Cycle 

**Figure 4:**
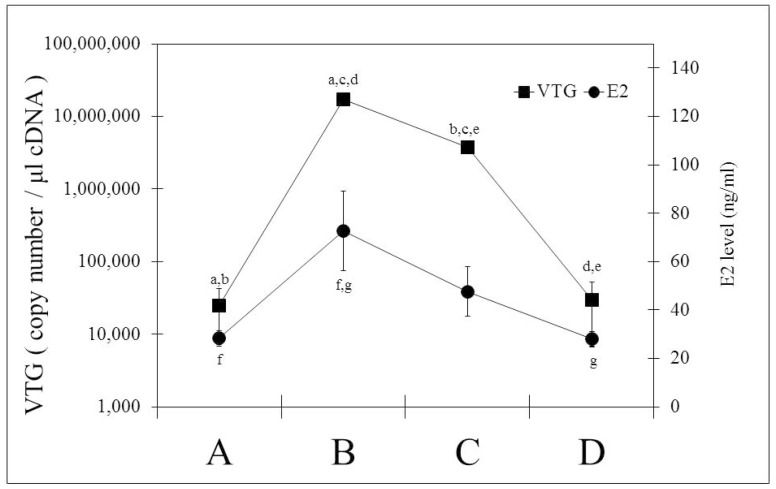
Physiological changes during the reproductive cycle of mature female rainbow trout from September to January: Alterations of the hepatic vitellogenin (*VTG*) mRNA levels of the liver and the serum 17β-estradiol (E2). Categorization of the fishes into maturation stages was done based on the gonadosomatic index (GSI) and the histological appearance of the oocytes; Stage A: fish at the beginning of ovarian development (GSI < 1), with primary follicles and partly cortical alveolar oocytes (*n* = 4), Stage B: fish with enlarged ovaries (GSI 12 < 18) and vitellogenic oocytes; additionally they possess an enlarged liver (liver somatic index LSI > 1.5) (*n* = 5), Stage C: Spawning fish with large ovaries (GSI > 18), mature oocytes and reduced liver size (*n* = 3), Stage D: Post-Spawning fish, with low (GSI < 5), spent follicles and a LSI close to 1 (*n* = 4). Statistically significant differences between groups are indicated by the same letter (a–g). The absolute gene copy number of *VTG* per 1 µL cDNA in the liver and E2 concentrations are shown as mean ± SE.

Changes of hepatic and immune *ER* gene transcript levels were studied in female rainbow trout over a full spawning cycle. The reproductive status of the fishes was assessed by measuring liver-somatic index (LSI), mRNA levels of hepatic vitellogenin (Figure 4), plasma E2 concentrations (Figure 4), and gonadosomatic index (GSI). Additionally, the ovaries were examined by histology to assess the maturation status of the oocytes. Based on these criteria, fish were categorised into four stages: Stage A—fish at the beginning of the reproductive cycle, with low LSI, a GSI less than 1, low hepatic vitellogenin mRNA levels, low serum E2 levels and immature and partly cortico-alveoloar oocytes; Stage B—vitellogenic fish, with enlarged liver (LSI > 1.5), increased ovaries (GSI 12–18), significantly elevated hepatic vitellogenin mRNA and serum E2 levels, and vitellogenic oocytes; Stage C—spawning fish, with high LSI, high GSI, significantly reduced serum E2 and hepatic vitellogenin mRNA levels, and mature oocytes; Stage D—post-spawning fish, with reduced LSI, low GSI (close to stage A), low vitellogenin mRNA, and low E2 levels, similar to stage A. The ovaries of stage D fish display spent follicles. 

**Figure 5:**
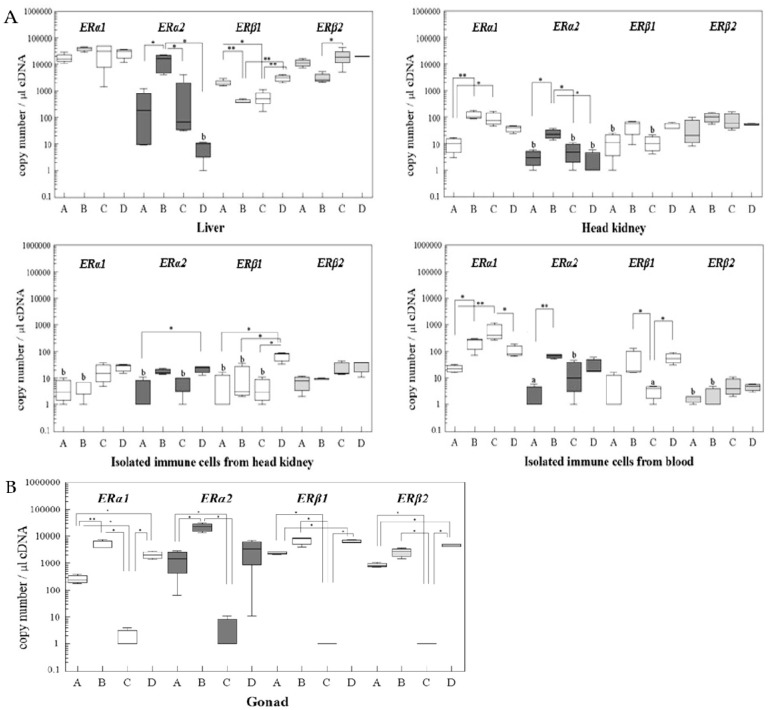
(**A**): The mRNA expression levels of the four ER isoforms in the liver, head kidney (HK), immune cells isolated from head kidney or from blood during the reproductive cycle. The reproductive cycle was subdivided into four stages: stage A = start of reproductive cycle, stage B = vitellogenic stage, stage C = spawning stage, stage D = post-spawning stage (see Figure 4). The gene copy number of each isoform gene per 1 µL cDNA is presented by Box-Whisker plots (group A: *n* = 4, B: *n* = 5, C: *n* = 3, D: *n* = 4). Note logarithmic scale of y-axis. * *p* < 0.05, ** *p* < 0.01. (**B**): The mRNA expression levels of the four ER isoforms in the ovaries during the reproductive cycle.

Figure 5 reports the mRNA changes of the four ER subtypes in the liver, gonads, and immune organs and cells of mature rainbow trout over the reproductive cycle, i.e., from stage A to stage D. In the liver, *ERα1* mRNA showed a slight tendency for increasing values with maturation and a decrease towards the post-spawning stage; however, the differences are not statistically significant. In contrast, hepatic *ERα2* mRNA experienced strong and significant changes during the reproductive cycle. With *ERβ1*, we observed a significant downregulation in the liver with increasing maturation of the fishes, and a partial recovery during the post-spawning stage. For the hepatic *ERβ* isoforms, alterations took place from stage A to C, with significant downregulation in the case of *ERβ1* and significant upregulation in the case of *ERβ2*. Thus, each of the four ER subtypes in the liver showed an individual pattern over the reproductive cycle, and the pattern was partly different to the changes of the hepatic ER profile of juvenile trout under E2 exposure. 

In the head kidney, mRNA levels of the *ERα2* isoform varied over the reproductive cycle similar to the behaviour of the *ERα2* isoform in the liver. In contrast to the liver, however, *ERα1* gene transcript levels experienced significant variations in the head kidney, whereas the *ERβ2* isoform remained unchanged. 

The reproductive cycle was associated with alterations of ER expression levels in the leukocytes. A significant mRNA upregulation of *ERα2* and *ERβ1* was observed in the head kidney leukocytes of post-spawning females, and also in the blood leukocytes, *ERβ2* gene copy numbers increased in the post-spawning females. *ERα1* gene transcript levels of blood leukocytes, however, decreased towards the post-spawning stage, after they had increased from stage A to C. 

In the gonads (Figure 5B), the most prominent response of the ER expression patterns during the reproductive cycle was the strong mRNA downregulation of the two *ERα* isoforms in reproductive stage C. The gene transcripts levels of the *ERβ* isoforms in the gonads showed limited variation during the reproductive cycle.

A regression analysis between the changes of serum E2 concentrations and tissue *ER* mRNA levels in mature rainbow trout yielded overall moderate to low correlation coefficients (Table 2). The strongest correlations to E2 were observed for *ERα2* and *ERβ1*. The *ERα* isoforms usually showed a positive correlation, i.e., mRNA increased with increasing E2 concentrations, whereas with the *ERβ* isoforms, also negative correlations were found. In general, the poorest *ER*-E2 correlation existed for the blood leukocytes. This could be due to a low estrogen sensitivity of the cells or to alterations in the cellular composition of the blood leukocyte population [41]. 

## 3. Discussion

To provide a baseline for understanding the physiological role of estrogens in the immune system of teleost fish, this study (1) characterized the mRNA expression levels and ratios of the four ER isoforms [40] in immune organs and cells of rainbow trout, (2) examined their response to exogenous or endogenous variations of estrogen concentrations, and (3) compared the mRNA levels of the ER isoforms in the immune system to that of the hepatic, and partly also, gonadal ERs. A first finding of this study is that the immune organs and immune cells of rainbow trout express all four ER isoforms, namely ERα1, α2, β1 and β2. Expression of nuclear ERs in immune cells is well documented for mammals, where both nuclear ER subtypes, ERα, and ERβ, are present in most immune cells and hematopoietic progenitor cells [4,9,10,42,43]. The differential expression of the ER subtypes in the immune cells influences gene regulation and appears to be important to balance the multiple effects of estrogens in the mammalian immune system [13,44,45]. Generally, the ERα subtype appears to have a more prominent expression and distribution in mammalian immune cells than ERβ [5]. Also, in the trout immune system, ERα is prominently expressed, but at least in the immune organs, head kidney and spleen, the *ERβ2* gene copy numbers are in the same range as those of *ERα*, pointing to an important role of this ER isoform in teleostean immune organs.

Presence of nuclear ERs in isolated immune cells has been assessed by means of relative mRNA quantification for a number of teleost species other than the rainbow trout: For seabream (*Sparus aurata*), Liarte et al. [27] reported no presence of nuclear *ER* gene transcripts in the testicular and head kidney acidophilic granulocytes, whereas macrophages and lymphocytes isolated from the head kidney contained *ERα* mRNA, but not *ERβ1* or *ERβ2* mRNA. For channel catfish (*Ictalurus punctatus*), Iwanowicz et al. [39] described expression of *ERα* and *ERβ* mRNA in primary leukocytes from head kidney and spleen, while only *ERα* was detected in peripheral blood leukocytes. For carp (*Cyprinus carpio*), Szwejser et al. [31] found high mRNA levels of *ERα,* but no *ERβ* gene transcripts in peripheral blood leukocytes. In leukocytes that were isolated from the head kidney of carp, *ERβ* could be detected although at very low levels. Thus, in all three species the tissue leukocytes displayed higher gene transcript levels of *ERα* than of *ERβ*, and the later was completely absent from peripheral blood leukocytes. In contrast to these studies, we detected both *ERβ* subtypes in the peripheral blood leukocytes of rainbow trout. Interestingly, however, while in the intact head kidney and spleen, the *ERβ2* mRNA numbers equalled those of *ERα1*, the isolated leukocytes displayed 10–100 times lower mRNA numbers of *ERβ2* than of *ERα1*. We found the two ER isoforms not only in blood leukocytes but also in head kidney leukocytes, together with *ERα1* and *ERα2*. Also Shelley et al. [30] reported the presence of mRNA of all four ER subtypes in head kidney leukocytes of rainbow trout. Thus, the overall picture arising from the various studies on nuclear ER in the immune system of diverse teleost species point to ERα/ERα1 being the dominant nuclear ER isoform in the immune cells, but not necessarily in the immune organs. The expression of ERβ in fish immune cells appears to vary with the origin of the cells and across species. 

Expression levels of *ERα1* in the immune organs of juvenile rainbow trout were significantly lower than in the liver. Also for *ERβ1*, the head kidney and the isolated leukocytes (but not spleen) displayed significantly lower mRNA levels than the liver, while no significant tissue differences existed for *ERα2*. Our findings agree with those of Nagler et al. [40] who identified the liver of rainbow trout to be the organ with the highest gene transcript levels of *ERα1* and *ERβ2* and clearly lower levels in immune organs. Similarly, Massart et al. [29] observed much higher *ERα1* mRNA levels in the liver of rainbow trout than in head kidney and spleen. However, at the protein level, Massart et al. [29] found no clear difference of the ERα expression in liver compared to head kidney and spleen. Discrepancies between ER levels at the protein and mRNA levels have been observed also in other studies, for instance, Pinto et al. [46] found no measurable *ERα* mRNA in the scales of sea bream scale, whereas ERα protein was well detectable. In this context it is important to keep in mind the complexity of ER regulation as it has been highlighted from recent studies with mammals [36,37,38,47]. The “classical” view of estrogen receptor activity is that, after binding of E2, ER dimerizes, and translocates into the nucleus where it binds to Estrogen-Response Elements (ERE) on target gene promotors to activate or repress transcription. However, there are a number of different regulation processes involved, including the cell-specific availability of co-repressors and co-activators, ER stability or proteolysis as well as post-translational modifications, such as ER phosphorylation. In addition, cross-talks with other signaling pathways such as the insulin-like growth factor 1 receptor pathway modulate the dynamics of ER-mediated gene regulation. Vice versa, both liganded and unliganded ERs are able to influence other signaling pathways. Altogether, these diverse processes of ER regulation and activity largely drive the target cell-specific estrogen actions. ER sequences influence isoform conformation, turnover rates and also the regulation by co-regulators, and thus can provide a basis to understand the E2 dependence of ER expression. Here, the information that the four ER isoforms of rainbow trout show similarity of their amino acid sequences, particularly in the AF1 and AF2 domains [40], is an important starting point for unravelling the mechanisms of ER functions in the trout immune cells. 

A striking difference in absolute *ER* gene copy numbers that was observed in this study existed between intact immune organs and the pure leukocyte preparations. *ERβ1* and *ERβ2* gene transcript numbers were significantly lower in the isolated leukocytes. Only for *ERα1*, the blood leukocytes had higher gene copy number levels than the head kidney and as a high levels as the spleen. Similar results have been reported by Iwanowicz et al. [39] for channel catfish. This suggests that the ERβ isoforms have a prominent function in the leukocytes. Blood and head kidney leukocytes differed in their ER expression profiles in what is likely to reflect a different cellular composition [31]. 

Taken together, the findings from this study provide evidence that immune organs and cells of rainbow trout express all four ER isoforms, although mostly at low levels, and that ER profiles of the immune organs and cells differ strongly to each other. With a relatively strong expression of ERβ2, the immune organs are more similar to the liver than to the leukocytes, which show a dominance of ERα1. 

A second aim of this study was to evaluate how *ER* mRNA levels in immune organs and cells of rainbow trout respond to changing E2 concentrations under different physiological conditions. This was investigated on one hand by exposing sexually immature juvenile trout to exogenous E2. At this life stage, the gonads of salmonids are already differentiated into ovaries and testes but endogenous sex steroid production is still negligible or very low [48,49]. Thus, elevating the estrogen concentrations of these animals by exposure to exogenous E2 was considered to represent a non-physiological situation. On the other hand, we examined mature female rainbow trout over a full reproductive cycle. In this situation, the endogenous alterations of E2 levels are embedded in a number of additional physiological changes, and thus, E2 is not acting in isolation, as in the juvenile fish, but in concert with other factors. We were interested to compare these two situations since differences of the physiological states can strongly influence the estrogenic regulation of ER expression [35,50,51].

The induction or suppression of the number of nuclear ER by E2 (autoregulation) is a way by which a target organ or cell can modulate its sensitivity to estrogens [34,35,52]. In mammals, ER autoinduction has been demonstrated for the liver and for reproductive tissues, as well as for immune cells. Molero et al. [53] showed that an increases of plasma E2 concentrations during the menstrual cycle of women are accompanied by an elevation of ERα and ERβ expression in the neutrophils. In contrast, in isolated neutrophils of males, E2 upregulated only ERα, but not ERβ. In human macrophages, E2 upregulated the expression of the ER splice variant, ERα46 [33]. In teleost fish, ER autoinduction has been described to date mainly for the liver [35]. For instance, Menuet et al. [54] reported that short-term exposure of mature zebrafish with E2 resulted in a strong upregulation of hepatic *ERα*, a marked reduction of the mRNA levels of hepatic *ERβ1* and virtually no change of *ERβ2*. Injection of male largemouth bass (*Micropterus salmoides*) with E2 led to a dose-dependent upregulation of hepatic *ERα*, but had no clear effect on the hepatic *ERβ* isoforms [55]. Comparable findings were reported from in vivo studies with fathead minnow (*Pimephales promelas*) [56], and from in vitro studies with isolated trout hepatocytes [57]. In the liver of male goldfish receiving E2 implants, *ERα* was highly upregulated, *ERβ1* was significantly downregulated and *ERβ2* did not change [58]. As summarized by Nelson and Habibi [35], estrogen-dependent upregulation of hepatic *ERα* appears to be fairly ubiquitous across species, whereas the estrogenic regulation of the hepatic *ERβ* isoforms varies strongly with species and experimental/physiological conditions. This is confirmed by the results of the present study: E2 exposure of juvenile trout led to significant mRNA upregulation of the two *ERα* isoforms but had no effect on *ERβ1* mRNA and significantly downregulated *ERβ2* mRNA.

Tissue differences in the response of the nuclear ER to estrogens are prominent. This has been demonstrated for mammals [59] and for fish as well [60]. Here, we focused on the regulation of the ERs in juvenile trout immune organs and cells by short-term (five days) exogenous E2 administration. The key finding is that exposure of sexually immature female rainbow trout to exogenous E2 concentrations that were sufficiently high to cause a significant vitellogenin mRNA induction did not lead to significant changes in the mRNA levels of all four ER isoforms, in the head kidney organ, in the head kidney leukocytes, or in the blood leukocytes. This behaviour is in contrast to the prominent responses of the hepatic ER. In another study with in vivo exposure of rainbow trout to E2, Shelley et al. [61] found an upregulation of *ERα1* mRNA in leukocytes from head kidney and blood, an upregulation of *ERα2* mRNA in head kidney leukocytes, but a downregulation in blood leukocytes, and no change of the gene transcript levels of the *ERβ* isoforms. Interestingly, in vitro exposure of rainbow trout blood leukocytes had no effect on the gene transcript levels of the four ER isoforms [61]. Developmental exposure of tilapia (*Oreochromis niloticus*) to ethinylestradiol was associated with elevated *ERα* gene transcript levels in the spleen, but not in the head kidney [26]. Finally, Liarte et al. [27] found an upregulation of *ERα* and *ERβ2* mRNA after in vitro treatment of specific macrophage cultures with E2. Given the variations of experimental conditions between the cited studies, as well as the species differences, it appears to be too pre-mature to come up with a general statement on whether ER autoregulation does exist in the immune system of fish or not. 

In the third part of the present study we examined how immune *ER* mRNA levels of mature female rainbow trout change with the reproductive cycle and the associated fluctuations of plasma E2 concentrations. In contrast to sexually immature fish, the immune ERs of mature fish experienced changes of their mRNA expression levels. This may indicate that the effect of E2 in the immune system is not a simple function of estrogen concentration, but depends on the overall physiological context [35,50,51]. One key finding from the analysis of the *ER* mRNA expression levels in the immune system of mature female rainbow trout is that the reproduction-related changes of ER isoform profiles in the immune tissues and cells are clearly different to the corresponding changes of ER profiles in liver and gonads. Even within the immune system, there exist distinct differences between the leukocytes from head kidney and those from blood. A second key finding that the reproduction-related changes of nuclear ER expression in the immune system are mainly restricted to the *ERα* isoforms, whereas the *ERβ* isoforms are less responsive. Also, while the *ERα* isoforms tend to increase with increasing E2 concentrations, *ERβ* isoforms tend to decrease if they respond at all. Finally, a third important observation is that the correlation between the plasma E2 concentrations and the immune *ER* gene transcript levels is overall moderate to low. 

The organ differences of the ER changes highlight again the importance of the specific cell and tissue environment for shaping expression and activity of the nuclear ERs [4,59]. The differences between the leukocyte populations of head kidney and blood are likely to reflect differences in their cellular composition. The head kidney population, in addition to differentiated immune cells, contains also diverse developmental stages of immune cells. Estrogens are master regulators of cell proliferation and differentiation and in line with this, ER are well expressed in developing immune cells of mammals. Importantly, the ER isoform profile of mammalian immune progenitor cells differs from that of mature immune cells [4,9]. If the situation is similar in fish, this may explain our finding of contrasting ER profiles between head kidney leukocytes and blood leukocytes of trout. 

The functional interpretation of the reproduction-related changing the *ER* mRNA profiles of the trout immune cells is difficult if not impossible at the current state of knowledge on the immune functional roles of the four isoforms. In mammals there exists evidence that the ERα subtype mediates anti-inflammatory actions in the immune system, [13,62], and the upregulation of this subtype by the elevated E2 levels during pregnancy is considered as one mechanism of the pregnancy-associated lowering of the immune activity in women. Likewise, the increase of immune ERα isoforms in trout with progressing ovarian maturation may represent an immunosuppressive mechanism as well. However, different to mammals, the purpose of this mechanism in oviparous fish could not be the protection of the embryos, but should have an alternative function, for instance, it may be speculated that it is mediating resource trade-offs between the immune and reproductive systems [63]. 

When initiating this study, we expected a rather close correlation between nuclear ERs in the immune system of rainbow trout and E2 levels, and we expected relatively high gene copy numbers of the *ER*s in the immune cells since E2 has prominent immunomodulatory actions in fish [21]. Our results prove the opposite to our expectations—the correlation between E2 levels and nuclear ER mRNA levels is moderate at its best, and the *ER* mRNA numbers in immune organs and cells are very low. The discrepancy between the pronounced immunomodulatory activity of estrogens in trout and low nuclear ER numbers and the limited estrogen-responsiveness suggests that the estrogen actions on the trout immune system involve, in addition to genomic signaling, alternative mechanisms. These could include membrane estrogen receptors [28,31], or indirect effects via interaction with other endocrine systems. Such indirect effects are well documented for the immune effects of estrogens in mammals [64,65,66,67], and may be of particular importance to mediate the resource trade-offs between the immune system and other fitness-relevant traits. 

In conclusion, the results from this study provide insight into the tissue-specific and physiological status-related expression and estrogenic regulation of the four nuclear ER isoforms in rainbow trout. While all four nuclear ER isoforms are present in the immune organs and immune cells of rainbow trout, their expression levels, ratios, as well as their autoregulation by E2, show distinct differences to liver or gonads. This data provides important baseline information for the immunomodulatory role of estrogens in fish, but to advance our understanding we need more insight into the functional role of the ER isoforms in the immune system, as well as an on the relative importance of genomic estrogenic signaling versus non-genomic and/or indirect pathways of estrogen action. 

## 4. Materials and Methods

### 4.1. Animal Experiments 

#### 4.1.1. Juvenile Rainbow Trout

Juvenile all-female rainbow trout (*Oncorhynchus mykiss*) of an average weight of three grams were bought at DSM SA (Village Neuf, France) and were reared at the Centre for Fish and Wildlife Health, University of Berne, Switzerland. Fish were kept at 11.3–11.8 °C, in 130 L flow-through glass tanks supplied with tap-water (approx. 1 L/m), constant aeration, and artificial light (12 h light to 12 h dark). On arrival, ten fish were randomly sampled and were screened for the presence of pathogens. No infectious agents were found. Any mortalities were recorded, and necropsied and investigated for the presence of parasites and other infectious agents. The fish were fed with a commercial dry pellet (Hokovit, Bützberg, Switzerland) with 1.5% body weight per day.

When the fish were six months old and had achieved an average weight of 50 g samples, the fishes were split into two groups: a control group that received the commercial diet and a 17β-estradiol (E2)-exposed group that received the commercial diet enriched with 20 mg E2/kg diet: this concentration was found to be sufficient to induce an estrogenic condition of juvenile trout in previous studies [11]. The feeding with the E2-enriched diet lasted for five days; the feeding level was 1% body weight per day both in the control and in the E2-exposed groups.

#### 4.1.2. Adult rainbow trout

Two-year-old rainbow trout of the breeding stock of the Centre for Fish and Wildlife Health were maintained in 1500 L tanks under flow-through conditions and light/dark cycles of Berne, Switzerland from September 2012 to January 2013. Water temperatures varied between 11 °C and 15 °C. The period from September to January covered the reproductive cycle of the fish, form the onset of ovarian maturation through the vitellogenic and spawning stage to the post-spawning stage (see Results). The fish were fed with the commercial diet at 0.5% body weight/day.

### 4.2. Preparation of Samples and Immune Cell Isolation 

Trout were euthanized in neutralized MS222, and liver, head kidney, spleen, ovary, and blood were sampled. All procedures were carried out according to the Swiss legislation for animal experimentation guidelines (Ethics Comitee Bern, approval date 31 August 2017, approval No. BE84/11). The blood was taken from the caudal vein. In addition to the tissue sampling, leukocytes were prepared from blood and head kidney. A thousand-fold dilution from blood or head kidney cell preparations was used to count the number of leukocytes using a Neubauer chamber. Moreover, serum was collected to determine plasma E2 concentrations by means of competitive enzyme-linked immunosorbent assay (ELISA).

For the immune cell isolation from the head kidney, the tissue was mechanically disrupted and passed through nylon nets with 250 µm and 125 µm nylon mesh, and the cells were collected in L-15 medium (Gibco) containing 10 IU/mL heparin. For the immune cell preparation that was isolated from the blood, the blood was diluted 10 times with L-15 medium containing 10 IU/mL heparin. The resulting cell suspensions from blood or head kidney were layered onto a Ficoll solution (Biochrom AG, Berlin, Germany) and were centrifuged at 400× *g*, 4 °C for 40 min. The immune cell fractions were collected in L-15 medium, washed repeatedly, and then adjusted to the appropriate different concentrations. 

### 4.3. RNA Extraction and Gene Expression Analysis

Isolated immune cells adjusted to 10^7^ cells were stored in 1 mL of TRIzol reagent (Sigma-Aldrich, St. Louis, MO, USA), homogenized. After adding 200 µL of bromochloropropane (Sigma-Aldrich, Buch, Switzerland), cell sample was mixed and centrifuged at 10,000× *g* for 15 min at room temperature. An aqueous phase of each cell sample was replaced by 500 µL of isopropanol and samples were stored at −80 °C until use. Tissue samples (approximately 5 × 5 × 5 mm) were kept in RNAlater (Sigma-Aldrich) at 4 °C overnight and were then stored −20 °C before use. Tissues were replaced in TRIzol reagent and homogenized, followed by the phase separation with bromochloropropane. The RNA precipitation with isopropanol and ethanol wash for both cell and tissue samples were performed and the resulting RNA was dissolved in nuclease-free water. After the digestion of resting DNA with RQ1 RNase-Free DNase (Promega AG, Dübendorf, Switzerland), 500 ng of RNA were reverse-transcribed to cDNA using GoScriptTM reverse transcriptase containing random primers, and dNTP as described in the manufacturer’s protocol (Promega AG) and total volume of cDNA was adjust to 25 µL. The TaqMan^®^-based real-time RT-PCR was carried out in triplicate for each sample mixture of total volume (12.5 µL) with 1 µL of cDNA template, 0.5 µM of each forward and reverse primer, 0.2 µM of the probe and TaqMan^®^ Gene Expression Master Mix (Applied Biosystems, Foster City, CA, USA) using a 7500 Fast Real-time PCR System (Applied Biosystems). The used primer and probe sequences were listed in Table 3. Expression of each ER isoform was calculated by absolute quantification using each plasmid DNA that prepared with a pGEM-T Easy Vector System I (for *ERα1* with fwd: 5′-CGGCCCCTCTCTATTACTCC-3′, rev: 5′-TGTACGACTGCTGCCTATCG-3′, for *ERα2* with fwd: 5′-TGCTGGTGACAACAGTGTCC-3′, rev: 5′-GGCCCAACTGCTGACTAGAA-3′, for *ERβ1* with fwd: 5′-CAGCTACCGGGGTCATAAAC-3′, rev: 5′-ACAGGCACAGGTCCACAAAT-3′, for *ERβ2* with fwd: 5′-TCATTCCAGCAGCAGTCATC-3′, rev: 5′-CTGAGGTACACATCTCCCCTCT-3′), and expressed mean of copy number per 1 µL cDNA ± standard error. In accordance with our PCR-system, the detection limit of *ERα*1, *α*2, *β*1, and *β*2 was 1, 5, 10, and 1 copy/µL cDNA, respectively. As an endogenous reference, 18S rRNA (Applied Biosystems, Foster City, CA, USA) was measured for the quality check of reverse-transcription of each cDNA. The gene expression level of liver-vitellogenin (VTG, Hamburg, Germany) [68] was utilized as an indicator for E2 response. 

### 4.4. In Situ Hybridization 

Plasmid DNA of ERs (*ERα1* with fwd: 5′-CTCTCCCCAGCCAGTCATAC-3′ and rev: 5′-CCTCCACCACCATTGAGACT-3′, *ERβ*1, and *β*2, as described above) was cloned in pGEM-T Easy Vector System I. Following digestion with NdeI and NcoI (Promega, Medison, MI, USA), linearized plasmid DNA was transcribed with T7 and SP6 polymerases (Roche Diagnostics AG, Rotkreuz, Switzerland), respectively, and labelled with digoxigenin (DIG) (Roche Diagnostics AG), as described in the manufacturer’s protocol. Synthesized labelled probes were stored at −20 °C in 50/50 (*v*/*v*) nuclease-free water/formamide buffer before use. 

Dissected organs, liver, and head kidney were placed immediately into cold Histochoice MB (Electron Microscopy Sciences, Hatfield, PA, USA) and were fixed at 4 °C for 3 h. Fixed organs were dehydrated in a graded ethanol series at 4 °C. For paraffin-embedding, the tissue were infiltrated with Histoclear (National Diagnostic, Chemie Brunschwig, Lausanne, Switzerland) for 60 min at room temperature, followed by Histoclear/Paraplast (50/50, *v*/*v*) for 60 min at 65 °C twice. After repeated cleaning in 100% of Paraplast for 60 min at 65 °C, tissues were incubated in 100% of Paraplast for overnight at 65 °C. The tissues were embedded in the fresh prepared Paraplast and stored at 4 °C before sectioning.

Tissues were deparaffinised and washed in diethyl pyrocarbonate (DEPC)-treated water. The acetylation of sections was performed in a buffer containing 100 mM of triethanolamine (pH 8.0) and 0.25% of acetic anhydride by shaking for 10 min. After repeated washing, hybridization was done using an antisense RNA- digoxigenin (DIG) probe in a hybridization buffer that was mixed with 50% deionized formamide, 4 × saline-sodium citrate (SSC), 10% dextran sulfate, 1 × Denhardt’s and 1 mg/mL ribonucleic acid from torula yeast for 16 h at 50 °C in a humid box. Sense RNA-DIG probe was applied in the same hybridization buffer as negative control. For post-hybridization, the slides were washed in tris-buffered saline with Tween20 (TTBS) (0.5 M NaCl, 0.1 M Tris-HCl (pH 8.0), 0.1% Tween-20). Following blocking with 6% milk powder that was diluted in TTBS for 1 h and bovine serum albumin (BSA)-Triton X-100 buffer containing 0.1 M Tris-HCl (pH 7.5), 0.15 M NaCl, 1% BSA and 0.3% Triton X-100 for 1 h, the specimens were incubated with a sheep anti-DIG antibody-alkaline phosphatase (AP) (Roche Diagnostics AG, Basel, Switzerland) diluted to 1:500 in the BSA-Triton X-100 buffer for 2 h at room temperature. The slides were then washed in the BSA-Triton X-100 buffer three times for 20 min. To equilibrate the slide, a buffer containing 0.1 M Tris-HCl (pH 9.5), 0.05 M MgCl_2_ and 0.1 M NaCl was used for 15 min, then the nitro blue tetrazolium (NBT)/5-bromo-4-chloro-3-indolyl-phosphate (BCIP) was applied on the slide for the development. The reaction was stopped by Tris-EDTA (TE)-buffer containing 0.01 M Tris-HCl (pH 7.5) and 1 mM EDTA (pH 8.0). For the head kidney, the same procedure as described for liver until post-hybridization was done; then, an additional endogenous peroxidase-blocking step with 1% of hydrogen peroxide was performed to account for the high endogenous alkaline phosphatase in the head kidney, Afterwards, the visualization was done as follows: The sections were blocked using 5% normal donkey serum (Jackson ImmunoResearch, West Grove, PA, USA) diluted in TTBS; this was followed by 30 min incubation with a sheep anti-DIG antibody diluted to 1:1000 in TTBS. Then, the sections were incubated with a donkey anti-sheep antibody (Jackson ImmunoResearch) diluted to 1:100 in TTBS, and after repeated washing a sheep peroxidase anti-peroxidase (PAP) soluble complex diluted to 1:100 with TTBS was applied. NBT-BCIP was used for visualization. 

### 4.5. Competitive Enzyme-Linked Immunosorbent Assay (Celisa) to Determine 17β-Estradiol Concentrations in Serum 

The blood samples were centrifuged at 3000× *g* for 15 min at 4 °C. 200 µL of serum were diluted in 300 µL of PBS (pH 7.4) and then extracted by adding 3 mL of diethyl ether, vortexing for 10 s 6 times, and centrifuging at 1800× *g* for 10 min at 20 °C. After the samples were frozen at −80 °C for 20 min, the organic phase was transferred into a new glass tube and were completely dried in a heat block at 30 °C for overnight prior to be resuspended in 200 µL of PBS. 

A high binding ELISA-plate (Greiner bio-one, Frickenhausen, Germany) was coated with a mouse anti-rabbit antibody (Sigma-Aldrich, 1:2000 diluted in PBS) for 24 h at 4 °C. After repeated washes with PBST (0.05% Tween-20), the plate was blocked with 1% of BSA-PBST for 12 h at 4 °C. Fifty µl of the sample, 50 µL of the estradiol- horseradish peroxidase (HRP) (Cal Bioreagents, San Mateo, CA, USA, 1:10,000 diluted in PBS) and 50 µL of a rabbit anti-estradiol antibody (Cal Bioreagents, 1:2500 diluted in PBS) were mixed and incubated for 2 h at room temperature. For the standard, first 17β-Estradiol (Sigma-Aldrich) was dissolved in ethanol, and then the same volume of 17β-Estradiol instead of the sample ranging from 0.36 to 40 ng/mL diluted in PBS was used. Following five washes with PBST for 5 min each, the ABTS^®^ Peroxidase Substrate (Kirkegaard & Perry Laboratories, Maryland, USA) was applied for the color development. The plate was measured at 405 nm by an EnSpire 2300 Multimode Plate Reader (Perkin Elmer, Waltham, MA, USA). 

### 4.6. Statistical Analysis 

Normal distribution and homogeneity of variances of qRT-PCR data from control and E2-treatment group (Figure 2A,B) were first individually estimated. For statistical analysis between control and E2-treatment group within the same gene expression analysis, Student’s *t*-test or Mann-Whitney’s U test were applied. Multiple comparisons between different maturation stages were performed by Kruskal-Wallis test, followed by Sheffè multiple comparison test. Results were considered statistically significant when *p* < 0.05.

## Figures and Tables

**Table 1:** The mRNA ratios of the four ER isoforms in liver, head kidney, spleen, head kidney leukocytes and blood leukocytes.

Organ	Liver	Head Kidney	Spleen	HK Leukocytes	Blood Leukocytes
Ratio	Ratio *ERα1* mRNA to Other Isoforms	Ratio *ERα1* mRNA to Other Isoforms	Ratio *ERα1* mRNA to Other Isoforms	Ratio *ERα1* mRNA to Other Isoforms	Ratio *ERα1* mRNA to Other Isoforms
*ERα1* mRNA	1	1	1	1	1
*ERα2* mRNA	994	10	30	10	450
*ERβ1* mRNA	14	1	2	10	110
*ERβ2* mRNA	0.7	0.5	1.1	10	110

The ratios are calculated by dividing the absolute gene copy number (mean value) of *ERα1* in the respective organ or cell type by the absolute gene copy numbers (mean values) of the other isoforms. For instance, a value like “*ERα2* mRNA = 994” indicates that in this organ there are 994 times more gene copy numbers of *ERα1* than of *ERα2*.

**Table 2:** Correlation coefficients (*r*^2^) between serum E2 concentrations and *ER* mRNA abundance in liver and leukocytes of female rainbow trout over the reproductive cycle.

ER Isoforms	Liver	Head Kidney Leukocytes	Blood Leukocytes
*ERα1*	0.138 ↑	0.039 ↑	0.044 ↑
*ERα2*	0.241 ↑	0.203 ↑	0.014 ↑
*ERβ1*	0.282 ↓	0.282 ↑	0.009 ↓
*ERβ2*	0.019 ↓	0.054 ↓	0.041 ↓

Linear regressions were calculated between serum E2 concentrations and mRNA numbers of the four *ER* isoforms in liver and leukocytes of adult rainbow trout from different stages of the reproductive cycle. ↑ positive correlation (*ER* gene transcript levels increase with increasing E2 concentrations); ↓ negative correlation (*ER* gene transcript levels decrease with increasing E2 concentrations).

**Table 3:** Primer sequences used for the gene expression analysis and related accession numbers.

Gene		Sequence (5′-3′)	Accession No.
*ERα1*	Forward	CCCCCCAAGCCACCAT	AJ242741
	Reverse	TGATTGGTTACCACACTCGACCTATAT	
	Probe	CATACTACCTGGAGACCTCGTCCACACCC	
*ERα2*	Forward	TCCTGGAGCACAGCAAAGC	DQ177438
	Reverse	TGATCTTGAGACGCCCTTCTC	
	Probe	CCTCAGGACAGTAGCAAGAACAGCAGCTTC	
*ERβ1*	Forward	GGAGCGAGCCAATCAAGGA	DQ177439
	Reverse	GCCATGATCCGGCCAAT	
	Probe	TCTGCCCCACAGTATTAACCCCGGA	
*ERβ2*	Forward	CAGCTCCTGCTGTAGACACTCAGT	DQ248229
	Reverse	GGATGTACTAATGCTCTCGAGTGTTT	
	Probe	TGCTAACATTCCAAAACCCAGAGGAGAGC	
